# Evaluation of Global trigger tool as a medication safety tool for adverse drug event detection—a cross-sectional study in a tertiary hospital

**DOI:** 10.1007/s00228-023-03469-5

**Published:** 2023-03-11

**Authors:** Ville Valkonen, Kaisa Haatainen, Susanna Saano, Miia Tiihonen

**Affiliations:** 1 School of Pharmacy, University of Eastern Finland, P.O.B 1627, 70211 Kuopio, Finland; 2 Kuopio University Hospital, Kuopio, Finland; 3 Department of Nursing Science, University of Eastern Finland, Kuopio, Finland; 4 Hospital Pharmacy, Kuopio University Hospital, Kuopio, Finland

**Keywords:** Global trigger tool, Adverse drug events, Medication safety, Polypharmacy

## Abstract

The objective of this study is to describe and analyze adverse drug events (ADE) identified using the Global trigger tool (GTT) in a Finnish tertiary hospital during a 5-year period and also to evaluate whether the medication module of the GTT is a useful tool for ADE detection and management or if modification of the medication module is needed. A cross-sectional study of retrospective record review in a 450-bed tertiary hospital in Finland. Ten randomly selected patients from electronic medical records were reviewed bimonthly from 2017 to 2021. The GTT team reviewed a total of 834 records with modified GTT method, which includes the evaluation of possible polypharmacy, National Early Warning Score (NEWS), highest nursing intensity raw score (NI), and pain triggers. The data set contained 366 records with triggers in medication module and 601 records with the polypharmacy trigger that were analyzed in this study. With the GTT, a total of 53 ADEs were detected in the 834 medical records, which corresponds to 13 ADEs/1000 patient-days and 6% of the patients. Altogether, 44% of the patients had at least one trigger found with the GTT medication module. As the number of medication module triggers increased per patient, it was more likely that the patient had also experienced an ADE. The number of triggers found with the GTT medication module in patients’ records seems to correlate with the risk of ADEs. Modification of the GTT could provide even more reliable data for ADE prevention.

## Introduction

Adverse events (AEs) and preventable errors are highly prevalent in healthcare and a major issue in patient safety [1, 2]. ADE are the leading cause of avoidable patient harm in health care and therefore an important target for patient safety improvement [3]. In order to improve patient safety and quality of care, it is crucial to recognize, validate, and understand where and why the problems actually occur [1, 2]. It is important to gather and analyze organization-specific data to recognize the locally relevant areas of improvement [4]. Traditionally, ADEs and errors have been identified through incident reporting, but this approach alone underestimates the actual prevalence of ADEs and errors as it relies on voluntary and active reporting [1, 2, 5–8]. Given the complexity of healthcare, none of the currently existing ADE and error detection methods are able to detect all the incidents alone.

Trigger tool methods enable investigation of patient records by using predefined “triggers,” which are used for screening data samples following a systematic process [2]. One of the most utilized trigger tool methods is the Global Trigger Tool (GTT) method developed by the Institute for Healthcare Improvement (IHI) [6, 8, 9]. GTT method is based on a retrospective review of a random sample of patient hospital records using triggers to identify AEs associated with patient care. The GTT consists of 47–55 triggers within different modules to identify potential AEs [2, 10]. As GTT method includes different, independent modules, it can be used as an improvement tool for specific patient safety themes. For example, the medication module of GTT is widely utilized to detect and evaluate ADEs [2, 6, 9]. When GTT is used to detect and review ADEs, the triggers in use are specific antidotes to drugs, abnormal laboratory results, and patient’s symptoms [6].

### Aim

The aim of this study was to describe and analyze ADEs identified using medication module of GTT in a Finnish tertiary hospital during a 5-year period. This study aimed to evaluate whether the medication module of GTT is a useful tool for ADE detection and management and whether there is an association between the current trigger set and risk to patient harm.

### Ethics approval

Research permission was obtained from the Kuopio University Hospital (KUH) in the autumn of 2021. Ethics approval was not required, according to the Finnish National Ethics Committee, because the research was based solely on de-identified registry data [11].

## Method

The GTT method has been applied systematically in KUH since 2014. KUH is a middle-sized tertiary hospital located in eastern Finland with about 450 beds and over 4000 personnel.

**Fig. 1:**
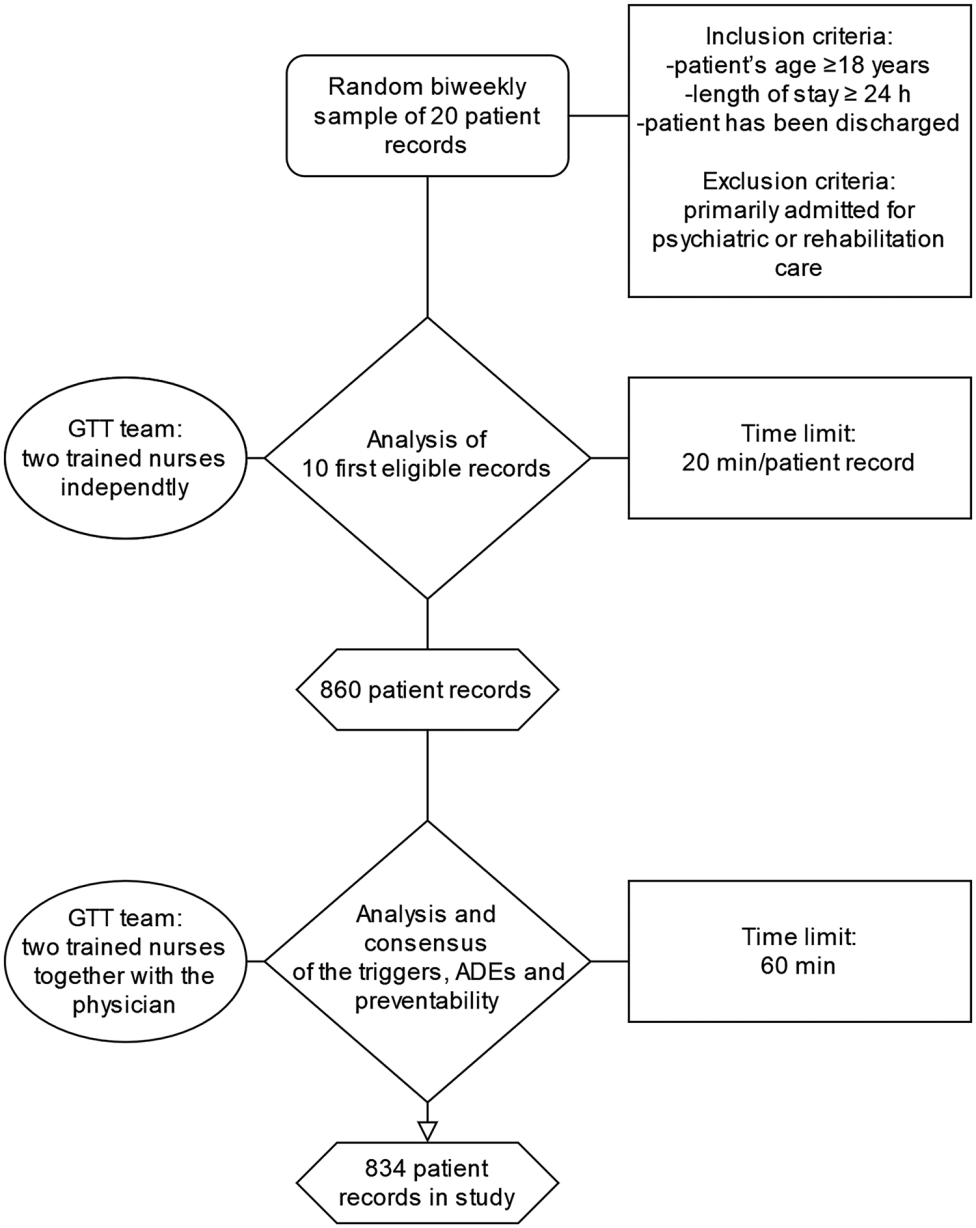
Study data collection with Global Trigger Tool in Kuopio University Hospital 2017–2021

The GTT method in KUH includes a bimonthly collected random sample of 20 patient records from electronic medical records using algorithms following predefined inclusion criteria described in Fig. 1. Even though the data sampling is based on predefined criteria, all the records in the random sample do not always meet the criteria. The GTT team members go through the records in the sample in the same order and review the first 10 records, which are eligible. The team consists of one physician and four trained registered nurses, which were selected through application and interview. In the review process, two trained nurses go through the patient records first independently and then together in a team meeting with the physician to form a consensus and analysis. In the GTT method, it is not intended to identify every single AE, and therefore the time to use in the review process is limited during the independent review to 20 min per patient record and in the GTT team meeting to a total time of 60 min to form a consensus of the analysis [2, 10].

A total of 834 records were reviewed by the GTT team in the years 2017–2021. In eight of the 86 meetings during the study period, the GTT team did not reach the goal of reviewing 10 patient records during the given time limit. Thus, the total number of patient records does not add up to the calculative number of biweekly samples in these years of study.

The GTT method in KUH is modified to include emphasized focusing on the patient’s point of view. In addition, the modified GTT included the evaluation of possible polypharmacy, NEWS, highest NI, and pain triggers, which are not included in the original GTT method [12, 13]. From these modifications, only polypharmacy was included in the analyses in this study. Polypharmacy was defined as the use of three or more regularly used medications in the modified GTT method of KUH [14].

The data analyses were conducted by using IBM Statistics 27 and Microsoft Excel for Microsoft 365 software. In this study, the total ADE rate as ADEs per 1000 patient days, ADEs per 100 admissions, and percentage of admissions with at least one ADE were calculated. ADEs were defined to be medication-related events that had evaluated to have caused harm to the patient. The severity of patient harm was categorized according to the National Coordinating Council for Medication Error Reporting and Prevention index (NCC MERP) [15]. Also, the preventable ADEs (pADE) were analyzed. The preventability of ADEs was determined by a consensus of the GTT team after thorough discussion and, if needed, after consultation with experts in the field of medicine in question. The positive predictive value (PPV) of each trigger was calculated by dividing the number of ADEs identified with that specific trigger by the total number of the same triggers found in the patient records.

This study also examined associations of polypharmacy and the number of medication module triggers to ADEs and length of hospital stay. Also, patient falls found with the triggers of other GTT modules were examined, as medication-related falls are known to be common [16–18].

In the statistical analysis, descriptive statistics were used to describe the characteristics. The Mann–Whitney *U*-test, *t*-test, or analysis of variance (ANOVA) were used for continuous variables and the chi-square test/Fisher’s exact test for categorical variables. Also, regression analysis was performed to determine the correlation of the variables. Results were considered statistically significant when *P*-value was less than 0.05.

## Results

In 2017–2021, the GTT team reviewed a total of 834 patient records (427 women and 407 men). The mean age for the patients was 63.5 years (range 18–105 years), and 601 (72.1%) patients had polypharmacy (three or more medications in regular use). The study data contained 366 (43.9%) patient records with triggers of GTT in the medication module. The trigger number ranged from 0 to 3 triggers per patient, and altogether, 448 (53.7%) medication module triggers were found. The GTT team evaluated that 53 (14.5%) of the 366 patients with medication module triggers had faced harm from the ADE related to the trigger finding, and of those, 32 (60.4%) would have been preventable as presented in Table 1.

**Table 1:** The study data characteristics by adverse drug event

**Variable**	**All patients**	**Patients without ADE**	**Patients with ADE**	**Patients with pADE**
Number (patients)	834	781	53	32
Age (years)	63.5 ± 17.4	63.3 ± 17.6	67.0 ± 14.0	63.1 ± 15.1
Gender (female)	427 (51.2%)	405 (51.9%)	22 (41.5%)	13 (40.6%)
Length of hospital stay (days)	5.3 ± 7.1	4.9 ± 5.9	11.0 ± 15.8	5.3 ± 7.1
Polypharmacy	601 (72.1%)	556 (71.2%)	45 (84.9%)	30 (93.8%)
*Medication module triggers per patient*				
0 triggers	468 (56.1%)	468 (59.9%)	0	0
1 trigger	295 (35.4%)	268 (34.3%)	27 (50.9%)	21 (65.6%)
2 triggers	60 (7.2%)	40 (5.1%)	20 (37.7%)	8 (25.0%)
3 triggers	11 (1.3%)	5 (0.6%)	6 (11.3%)	3 (9.4%)

The numbers are presented as *n* (%) or mean ± standard deviation

*ADE* adverse drug event, *pADE* preventable adverse drug event, *Polypharmacy*, regular use of three or more medications

The number of ADEs/1000 patient days was 13.0, and altogether 6.4% of all admission reviewed experienced at least one ADE during the hospital stay. ADEs/100 admissions were 6.83.

Altogether there were 57 ADEs identified with the medication module trigger tool in the study records. Most of the triggers found with GTT medication module were related to the use of antiemetics (42.4%), abrupt medication stop (36.8%), or oversedation/hypotension (8.5%), as seen in Table 2. The highest PPVs in predicting ADEs were found in trigger groups with blood glucose < 3–5 mmol/l (1.00) and other medication related (0.96).

**Table 2:** Adverse drug events (ADE), preventable adverse drug events (pADE), and positive predictive value (PPV) of medication module triggers

**Trigger**	**Triggers**	**ADEs**	**pADEs**	**PPV**
Antiemetic administration	190	3	2	0.02
Abrupt medication stop	165	5	4	0.03
Oversedation/hypotension	38	18	3	0.47
Other medication related*	23	22	21	0.96
Antihistamine administration	13	0	0	0
Rising urea or creatinine	6	2	0	0.33
Vitamin K administration	5	1	1	0.20
Clostridium difficile positive stool	4	3	0	0.75
PTT > 100 s**	2	1	1	0.50
Blood glucose < 3–5 mmol/l	2	2	0	1.00
Naloxone administration	2	0	0	0
Flumanzenil administration	1	0	0	0
INR > 6***	0	0	0	NA
**Total (*n*)**	**448**	**57**	**32**	-

* Adverse drug events discovered spontaneously without triggers; ** partial thromboplastin time; *** international normalized ratio

ADEs in the other medication-related trigger group contained adverse reactions relating to drugs such as dizziness and optical illusions, but also process-related medication errors such as omissions, improper dosing, wrong administration rate, wrong medication, and delayed administration. These are not included in the original GTT, but these were recorded and classified as other medication-related triggers when identified spontaneously during the GTT process.

**Fig. 2:**
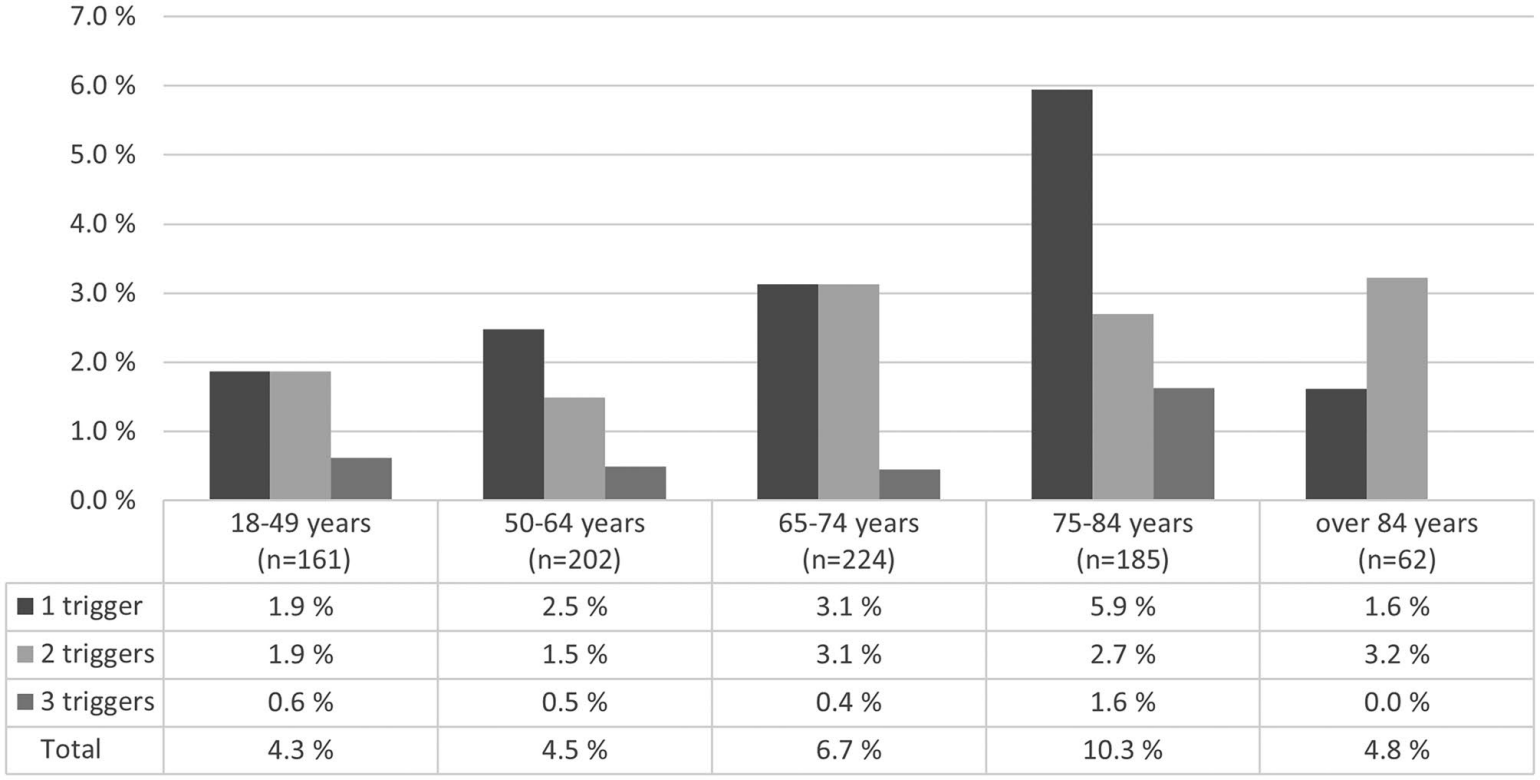
Prevalence of patients with adverse drug events in age groups by number of medication module triggers

Over 60% of ADEs were found in patients in the age group of 65–84 years, which is a bit higher portion than the age group itself (49.0%). The ADEs were slightly more common in the age groups covering ages 65–84 years than in other age groups, as shown in Fig. 2. However, this difference was not statistically significant (*p* = 0.152).

**Fig. 3:**
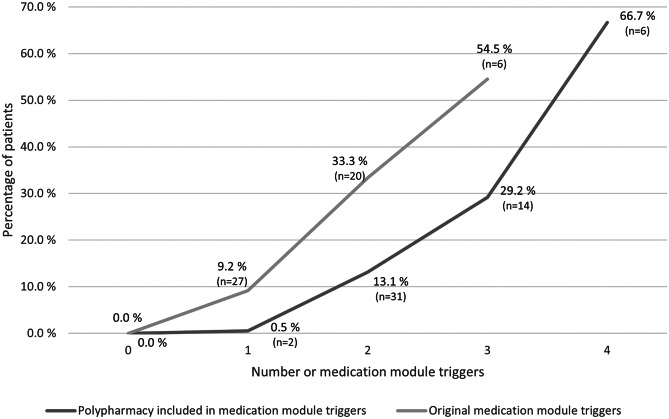
Prevalence of patients with adverse drug events by medication module triggers

The prevalence of ADEs was higher when the number of triggers found with the medication module increased, as shown in Fig. 3. The increase in the ADE prevalence by the trigger number was found statistically significant (1 trigger: 9.2%; 2 triggers: 33.3%; 3 triggers: 54.5%; *p* < 0.001). Also, the correlation between ADE prevalence and trigger number was statistically significant (*p* < 0.001). Altogether, 27 (9.2%) patients with one trigger, 20 (33.3%) patients with two triggers, and six (54.6%) patients had faced ADE during the hospital stay. The number of triggers was found to correlate also with the length of stay, as seen in Table 3. The correlations were found statistically significant (*p* = 0.000–0.022). When polypharmacy was included as one of the triggers in the medication module, the ADE prevalence was increasing even more strongly along with the number of triggers.

**Table 3:** Length of hospital stay and adverse drug events (ADE) by polypharmacy and the number of medication module triggers

	***n***	**Length of stay (days)**	**Patients with ADEs**	
**Patient grouping**		*Mean* ± *standard deviation*	*No ADE* *n (% of patients)*	*ADE* *n (% of patients)*
All patient records	834	5.3 ± 7.1	781 (93.6%)	53 (6.4%)
*Polypharmacy*				
No polypharmacy	233	4.9 ± 5.9	225 (96.6%)	8 (3.4%)
Polypharmacy	601	11.0 ± 15.8	556 (92.5%)	45 (7.5%)
*Triggers per patient*				
0 triggers	468	3.8 ± 3.5	468 (100%)	0
1 trigger	295	6.1 ± 8.4	268 (90.8%)	27 (9.2%)
2 triggers	60	8.9 ± 8.2	40 (66.7%)	20 (33.3%)
3 triggers	11	25.2 ± 22.8	5 (45.5%)	6 (54.5%)

*Polypharmacy*, regular use of three or more medications

Altogether, 72.0% (601 patients) of the patient records had polypharmacy. Also, 75.1% of the patients with at least one medication module trigger had as well polypharmacy. There was a statistically significant difference (*p* = 0.031) in patients with and without polypharmacy and the risk of ADE during the hospital stay, as seen in Table 3. The difference was noticeable also with preventable ADEs in polypharmacy groups (*p* = 0.005). Patients with polypharmacy had also significantly longer stay in the hospital, as the mean length of stay was 11.0 days for patients with polypharmacy and 4.9 days for patients without polypharmacy (*p* < 0.001).

This study found 14 patients with fall as a trigger and an AE by using the GTT method. Most of these patients (78.6%, 11 patients) had also polypharmacy as a trigger and also at least one trigger from medication module (71.4%, 10 patients). Difference of falls in patients with or without medication module trigger was found statistically significant (p = 0.036). The medication module triggers of patients with falls related to antiemetic administration, oversedation/hypotension, abrupt medication stop, and other medication-related triggers. From the falls, eight were classified to harm class E: temporary harm to the patient and requiring some intervention, and seven to harm class F: temporary harm to the patient and requiring initial or prolonged hospitalization.

## Discussion

In this cross-sectional study on Finnish tertiary hospital, we aimed to describe and analyze ADEs identified using the medication module of GTT during a 5-year period. This study found the GTT to be a useful tool in ADE detection and management. The prevalence of ADEs was found to correlate between the number of medication module triggers. The correlation was also found with the number of medication module triggers to the length of hospital stay. This finding provides useful information and a new perspective for clinical ADE detection and prevention, as it seems reasonable to also regard the total number of triggers per patient in addition to the trigger-specific findings. However, even though the ADE prevalence was correlated to the number of medication module triggers in this study, it was lower than many other GTT studies focusing on ADEs. In this study, the rate of ADEs/100 admissions was 6.8 and 13.0 ADEs/1000 patient days. In previous GTT ADE studies, these rates ranged between 6 and 61.3 ADEs/1000 patient days and 5–38.9 ADEs/100 admissions, respectively [9, 19, 20]. One possible explanation to lower ADE prevalence rates can be that studies with GTT method focusing on only ADEs can produce spontaneous, non-triggered ADEs more effectively than general GTT method [9]. Even though this study focused on the GTT medication module triggers and ADEs, the data was collected as a part of the routine GTT method in KUH, covering all the modules in the time frame. It is also challenging to compare the study results as there have been modifications present in the trigger sets and methodology in the previous studies. Thus, the inter-reliability of the GTT method has been raised as a concern in previous studies [21–24]. The reliability concerns are related mostly to the consistency of the review process and evaluation views among different GTT teams. In KUH, the GTT team has remained unchanged, which can be regarded as a strength from the perspective of consistency of the results. In our study, 6.4% of patient records were reviewed to have faced at least one ADE during the hospital stay, which is quite similar result as 10% found in a previous study evaluating KUH GTT data [14]**.** It is possible that different teams using slightly different protocol and point of view could detect variable prevalence rates for ADEs as well. More research is needed to confirm and evaluate this relationship.

Some medication module triggers, such as administration of antihistamine, flumanzenil, or naloxone, produced PPV valued zero. The trigger set included a trigger of INR > 6, which was not detected even once in the study data set. The modification of the INR trigger to discover lower overdosing should be considered the current laboratory value limit of this is set relatively high. The ideal INR value for patients using warfarin is 2–3, depending on the indication, so even lower values than 6 could indicate increased risk of bleeding [25–27].

Quite a few ADEs were also detected spontaneously and therefore classified as other medication-related ADEs. The preventability of ADEs was the highest in this group. Therefore, modification of the trigger set by analyzing the spontaneously detected ADEs in more detail could provide more useful insight into ADE prevention. Traditionally, GTT utilizes random samples of patient records, but modifying the perspective to analyze a specific subgroup of patients or organizational characteristics could provide new evaluation opportunities [28]. In addition to implementing new triggers, such as polypharmacy, also removing triggers that have never resulted in ADEs could enhance the clinical relevance and usability of the tool [29]. This requires careful planning and reflection on collected data so that potentially useful triggers are not removed too lightly as they have not yet resulted in ADEs. It is also important to assess whether the sample size is sufficient to represent the entire patient population. Instead of using standard GTT protocol in every organization, adjusting the sampling size and frequency to the size and type of the organization could provide more specific information [30]. Customization of the whole GTT process enables to meet specific needs of varying patient populations in different settings [31].

To our knowledge, this is the first study evaluating the use of polypharmacy in GTT to review the risk of ADEs and length of stay. In this study, polypharmacy was found to correlate with the risk of ADEs, pADEs, and longer stays in the hospital. Thus, polypharmacy could be a useful addition to the GTT medication module trigger set for ADE detection and management to prevent patient harm. In this study, polypharmacy was defined as regular use of three or more medications, which relatively rare but not unique definition [32]. However, modification of the polypharmacy trigger should be considered. Instead of using a binary trigger, the real number of medications or using a categorization to indicate the level of polypharmacy could be considered to enhance the accuracy of the trigger. The categorized polypharmacy trigger could, for example, include a categorization of minor polypharmacy (2–4 medications), moderate polypharmacy (5–9 medications), and excessive polypharmacy (10 + medications) [32]. Of course, it is important to bear in mind that, as with other triggers that do not always result in ADEs, neither does the increasing number of medications in regular use automatically mean ADE or inappropriate pharmacotherapy. One perspective could also be to record the active substances or therapeutic groups involved in ADEs for more thorough analysis of the risk medications. Polypharmacy can be defined in many ways, and in future, it would be interesting to study how different levels and rationality of polypharmacy correlates with the risk of ADEs and the length of hospital stays in GTT methodology.

The medication module triggers of patients with falls were related to antiemetics administration, oversedation/hypotension, abrupt medication stop, and other medication-related triggers. Thus, the study results seem to highlight well-known medication-related risk factors of patient falls, but more research is needed to confirm and understand this relationship [16–18]. Also, the other modified triggers, such as NEWS, NI, and pain triggers, would be interesting targets for further research.

This study has also some limitations. First, GTT is limited to detecting specific types of ADEs [5]. GTT is ineffective for detecting medication errors, potential adverse events, and errors in the dispensing and administration process, as they rarely are documented in patient records. Once implemented, the routine review work of GTT is a useful method to find and categorize ADEs [33]. The current GTT method still requires manual work as the GTT team must read through the medical record samples when searching the triggers, but automated trigger detection could enhance the efficiency of the GTT method [34]. Automation is found valid compared with traditional GTT method, and with further development, it could even possibly be used to detect the triggers in all electronic patient records instead of small data samples. This would transform the perspective from traditional retrospective GTT analysis to real-time medication safety and clinical outcome improvement by identifying and flagging the inpatients with high risk of AEs and ADEs during the hospital stay. Another future research topic could be automated analysis of the ADEs and errors by using natural language learning and artificial intelligence (AI), which have shown promising results in studies assessing the contents of incident reports [35].

## Conclusion

The number of triggers found with GTT medication module in patient’s record seems to correlate with the risk of ADEs. Also, new triggers, such as polypharmacy or organizational and patient group-specific modifications, could provide more reliable ADE information.

GTT provides useful insight into ADEs and should be considered to use as a part of portfolio of medication safety tools. However, it is crucial to understand the strengths and limitations of the GTT and apply the method to clinical practice accordingly to maximize its potential in medication safety improvement.

## Data Availability

Due to the nature of data from electronic medical records, it is not openly available. The datasets generated during and analyzed during the current study are available from the corresponding author upon reasonable request.

## Materials availability

Due to the nature of data from electronic medical records, it is not openly available. The datasets generated during and analyzed during the current study are available from the corresponding author upon reasonable request.
